# Recommendations for Improving Consistency in the Radiation Fields Used During Testing of Radiation Detection Instruments for Homeland Security Applications

**DOI:** 10.6028/jres.118.014

**Published:** 2013-05-28

**Authors:** L Pibida, M Mille, B Norman

**Affiliations:** 1 National Institute of Standards and Technology, Gaithersburg, MD 20899; 2 Nuclear Engineering and Engineering Physics Program, Rensselaer Polytechnic Institute, Troy, NY 12180

**Keywords:** ANSI/IEEE standards testing, exposure rate constants, radiation field determination, source activity

## Abstract

Several measurements and calculations were performed to illustrate the differences that can be observed in the determination of exposure rate or ambient dose equivalent rate used for testing radiation detection systems against consensus standards. The large variations observed support our recommendation that better consistency in the test radiation fields can be achieved by specifying the source activity and testing distance instead of the field strength.

## 1. Introduction

Commercially available radiation detection instruments for homeland security applications are currently being tested against American National Standard Institute/Institute of Electrical and Electronics Engineers (ANSI/IEEE) and International Electrotechnical Commission (IEC) standards. Table 1 lists the relevant ANSI/IEEE and IEC standards which have been published or are currently under development. These standards cover a wide variety of instruments from small personal radiation detectors (PRDs) to large radiation portal monitors (RPMs). The chief function of these detectors is to measure the magnitude of the radiation field (often expressed as exposure rate or ambient dose equivalent rate), and not to provide a dose record to the user. In fact, some of the instruments only report the radiation field in units of counts per second.

Most of the standards in Table 1 place specific requirements on the strength of the radiation field used for testing, typically defining it at the reference point of the detector through a measure of exposure rate (expressed in µR/h)^1^ or ambient dose equivalent rate (H^*^(10), expressed in units of µSv/h). Several of the tests described in these standards require fields ranging from 5 µR/h to 50 µR/h above average background levels at the test location. Yet, the background radiation for testing laboratories within the United States can range from 5 µR/h to 25 µR/h depending on the geographic location. This means that laboratories performing testing against these standards are required to determine the testing radiation field very close to or even below background radiation levels. This poses a challenge in the method used to determine the testing radiation field. Currently some of these standards suggest the use of an ion chamber or similar radiation detection device to determine the testing radiation fields.

The testing of radiation detectors against these standards is mainly performed by determining the radiation fields of point sources or small extended sources. In order to produce the desired field, each testing laboratory might then position their radioactive source closer to or further from the detector, depending on the available source activity. Furthermore, the source encapsulation or container will affect the emission of the low energy x-rays and gamma-rays, which in turn affects the measured or calculated exposure rate value at a fixed distance. In addition, instruments might not have a flat energy response for photons from 1 keV to 3 MeV and are principally calibrated only for ^137^Cs. In general, corrections must be made to instrument response before the devices can provide accurate readings for sources containing a different radionuclide species (i.e. more gamma lines with different energies than ^137^Cs). Furthermore, testing of radiation detectors can be conducted in different laboratories located in different geographical locations with different levels of background radiation. These laboratories may also have different types of instruments or ways of calculating the exposure rate or ambient dose equivalent rate used for testing. The combined effect of all these differences becomes particularly problematic when they can influence the outcome of the radiation detector test, causing it to pass or fail a requirement listed in an ANSI/IEEE or IEC standard. Therefore, it is critical to have a consistent way of setting up the radiation fields so that the instruments can be tested in a fair and equitable manner. To accomplish this, we recommended that the source activity or source gamma-ray emission rate (for a specific gamma-ray line) and measurement distance be defined as the testing parameters in the ANSI/IEEE and IEC standards instead of making use of ambient dose equivalent rate or exposure rate.

In this work we provide results which illustrate the expected variation when determining the testing radiation fields using different calculation and measurement approaches. The large variations observed support our claim that better consistency in the test radiation fields can be achieved by specifying the source activity or source gamma-ray emission rate (for a specific gamma-ray line) and distance instead of the field strength.

## 2. Measurements

The gamma ambient dose equivalent rate and exposure rate were measured using the Victoreen 451P-DE-SI-RYR^2^ (ambient dose equivalent rate), the Thermo FH40G-L (exposure rate), and the Ludlum 9DP (exposure rate), respectively. The measurements were performed in both the count rate and integration modes of the Victoreen 451P-DE-SI-RYR, the Thermo FH40G-L, and the Ludlum 9DP. The integration time was 300 s. Ten independent readings were acquired, from which the mean and standard deviation were calculated for each radionuclide. The Victoreen 451P-DE-SI-RYR is a pressurized 230 cm^3^ volume air ionization chamber, pressurized to 6 atmospheres with a plastic casing. The Thermo FH40G-L has an internal proportional detector with a plastic casing. The Ludlum 9DP is a pressurized 230 cm^3^ volume air ionization chamber, pressurized to 8.5 atmospheres with a plastic casing.

The sources used for these measurements are listed in Table 2. Each source was encapsulated with 0.254 mm of stainless steel. The construction of these sources is described in detail in Refs. [1] and [2].

Measurements were performed with each source placed at a distance of 1 m from the reference point of the instrument. The sources were placed in a polymethyl methacrylate (PMMA) holder that provides no additional shielding to the source. The source holder is mounted on a track system that can set the source-to-detector distance with a precision of 0.1 mm. The track system is placed on a laboratory bench located in a low scatter room. The source center was placed at a height of approximately 60 cm from the top surface of the laboratory bench.

The calibration of the Victoreen 451P-DE-SI-RYR, the Thermo FH40G-L and the Ludlum 9DP was verified by using one of the National Institute of Standards and Technology (NIST) ^137^Cs calibration beams. The instrument readings were within 8 % of the NIST delivered radiation field.

## 3. Calculations

Exposure rates for each radioactive source listed in Table 2 were calculated using two different methods: (1) Hand-calculation using a point-source approximation [3]; (2) Monte Carlo radiation transport simulation. Details about these two methods are described in the sections below. Both calculation methods used the same x-ray and gamma-ray emission probabilities published by the Laboratoire National Henri Becquerel (LNHB) [7] and the Brookhaven National Laboratory Nuclear Data Center (BNL) [8].

The exposure rate calculations were performed first by assuming unshielded radionuclide point sources in air, and then by including the 0.254 mm stainless steel encapsulation. The bare source values were compared with the published values by Smith *et al.* [9] and with those obtained from the Rad Pro calculator [10]. For the bare sources, the exposure rate constants were also calculated as a function of the photon cutoff energy to demonstrate the sensitivity of this quantity to the choice of emission lines included in the calculations.

The ANSI/IEEE standards also specify masking or simultaneous radionuclide identification tests as a function of the ratios of the individual radionuclide exposure rates. In these tests sources such as ^131^I or ^99m^Tc (medical isotopes) are required to be shielded by 7.64 cm of PMMA to simulate attenuation by the human body. The exposure rates per unit activity for this scenario were calculated using both methods at a distance of 1 m.

### Calculation Method 1: Point Source Approximation

The point source approximation method is described in Ref. [3] and can be summarized as follows. The exposure rate constant expressed in units of R m^2^ h^−1^ Ci^−1^ for an isotope that emits one photon of energy hν per disintegration is approximated as:

$$\Gamma_{\delta }=194.5\;{h\nu (\mu }_{\text{ab}}{/\rho )}_{\text{air}}$$

where hν is the energy of the photon emitted expressed in MeV and (µ_ab_/ρ)_air_ is the mass energy absorption coefficient for dry air expressed in m^2^/kg [4]. This equation assumes that the average energy required to cause one ionization in air is a constant equal to 33.85 J/C (eV per pair) and a cut-off energy of δ. In this paper the exposure rate constant is expressed in units of R m^2^ h^−1^ Ci^−1^ (non-SI units) because all the ANSI/IEEE standards listed in Table 1 specify exposure rate in units of R h^−1^ and activity in Ci (where 1 C/kg = 3876 R, 1 Ci = 3.7 × 10^10^ Bq).

The exposure rate constant for an isotope that emits photons hν_1_, hν_2_, hν_3 …_ hν_n_ and the number of these per disintegration is N_1_, N_2_, N_3_…N_n_ can be approximated as:

$$\Gamma_{\delta }=194.5\sum_{i}^{n}N_{i}h\nu_{i}{\left(\frac{\mu_{\mathrm{ab}}^{i}}{\rho }\right)}_{\mathrm{air}}.$$

The exposure rate, $\dot{X}$, at a distance *d* from a point source with activity *A* can then be expressed as:

$$\dot{X}=\frac{\Gamma_{\delta }A}{d^{2}}.$$

A narrow-beam of monoenergetic photons with an incident intensity *I*_0_, penetrating a layer of material with thickness *x* (expressed in cm) and density*ρ* (expressed in g/cm^3^), emerges with an intensity *I* given by an exponential attenuation law:

II0=exp[−(μρ)xρ]

where *μ/ρ* is the mass attenuation coefficient expressed in units of cm^2^/g [4]. Then for a shielded source the exposure rate can be approximated as:

X˙=A194.5∑i=1nNihνi(μabiρ)airexp[−(μρ)ixρ]d2.

### Calculation Method 2: Monte Carlo Radiation Transport

Monte Carlo radiation transport calculations were performed using the Monte Carlo N-Particle eXtended code version 2.5.0 (MCNPX) [5]. Each radionuclide source was modeled as a point source in air emitting photons with the appropriate energies and relative emission probabilities. MCNPX’s F6 tally was used to obtain a track-length estimate of the collision kerma in a 1 cm thick spherical shell of air of radius 1 m which was concentric with the source. The tally result (expressed in units of MeV/g/photon emitted) was then converted to exposure rate at 1 m per unit activity of the source (expressed in R h^−1^ Ci^−1^) through the equation:

$$\frac{\dot{X}}{A}=2.4436\times 10^{6}\cdot TN_{total}$$

where T is the MCNPX F6 tally result and *N_total_* is the total number of photons emitted per disintegration, which is calculated for each radionuclide as:

$$N_{total}=\sum_{i=1}^{n}N_{i.}$$

Like the hand calculation method described above, this equation assumes 33.85 J/C of charge released in air. The Monte Carlo simulations were performed in coupled photon-electron transport mode (mode p e) with the cutoff energies for both particle types set to the default value of 1 keV. MCNPX’s standard physics models were employed with their default settings and included photoelectric absorption, coherent and incoherent scattering, pair production, fluorescence, and bremsstrahlung generation (from electrons liberated by photon interactions). The photon and electron cross-sections were taken from the default MCLIB04 [6] and ELO3 tables, respectively. The stainless steel and PMMA shielding in the MCNPX simulations were modeled as a sphere of the given radius centered at the point source. One hundred million particle histories were simulated to ensure that the relative Monte Carlo statistical uncertainties were in all cases smaller than 0.1 %.

## 4. Results and Discussion

The calculated exposure rate constants, *Γ_δ_*, for several bare radionuclides of interest for ANSI/IEEE standards testing are summarized in Table 3 together with the values obtained from Refs. [9] and [10]. The point source calculations are given for two cut-off energies, 1 keV and 40 keV. MCNPX results are shown for two different decay data libraries from which the emission probabilities for all x-rays and gamma-rays with energies greater than 1 keV were extracted.

In Table 3 the MCNPX and the point source method calculations for ^226^Ra, ^232^Th and ^232^U include the entire decay chain and assume that the ^226^Ra and ^232^Th sources are in equilibrium, and that the ^232^U source is 15 years old. The values for these radionuclides listed in Smith *et al.* [9] do not include the progeny. However, Smith *et al.* [9] did combine decay schemes for those radionuclides which do not have photon emissions themselves, but which are in secular equilibrium with photon-emitting products (e.g. ^137^Cs/^137m^Ba). Smith *et al.* [9] used a cut-off energy of 15 keV and included all photons with emission probabilities greater than 0.01 %. Their calculations also neglected bremsstrahlung, assumed 34 eV per ion pair created in air, and the mass-energy absorption coefficients for air were obtained from [4]. On the other hand, the Rad Pro calculator included the entire decay chain for ^226^Ra, but not for ^232^Th or ^232^U. The Rad Pro Calculator assumes that the average amount of energy required to produce an ionization event in air is 33.8 eV per ion pair and restricts the calculations to photons with emission probabilities greater than 1 %.

From Table 3 it can be observed that the exposure rate constants differ depending on the photon emission probabilities, the cut-off energy, the mass-energy absorption coefficients, and the selected value for the average energy required to cause one ionization in air. The mass-energy absorption coefficients will depend not only on the choice of the atomic cross-section library, but also on the method used to interpolate the tabulated data to obtain values at the required photon energies. The calculated exposure rate constants display a variation between 1 % and 20 %, but for several radionuclides the variation was 50 % or larger.

The background subtracted exposure rate values measured for the sources listed in Table 2 using the Victoreen 451P-DE-SI-RYR, the Thermo FH40G-L, and the Ludlum 9DP are summarized in Table 4. For comparison purposes these values are listed together with the corresponding calculated values using the point source approximation (with a cut-off energy of 40 keV) and MCNPX method (using the LNHB decay library). The calculated values account for the 0.254 mm thick stainless steel source encapsulation. The ambient radiation background measured by the Victoreen 451P-DE-SI-RYR was (0.046 ± 0.027) µSv/h when measured in the rate mode and (0 ± 0.05) µSv/h when measured in the integration mode (300 s integration time). The ambient radiation background measured by the Thermo FH40G-L was (8.61 ± 0.37) µR/h when measured in the rate mode and (10.4 ± 0.5) µR/h when measured in the integration mode. The ambient radiation background measured by the Ludlum 9DP was (8.78 ± 1.61) µR/h when measured in the rate mode.

The reported uncertainties for the measurements are equal to the standard deviation of 10 readings with a coverage factor of k = 2. From Table 4 it can be observed that the ^137^Cs measurements, in the rate and integration mode for Victoreen 451P-DE-SI-RYR, Thermo FH40G-L and Ludlum 9DP detectors, agree within the uncertainty values, while the calculated values are within 30 % of the measured values. For all other radionuclides the variations between the measured and calculated values are much larger than the measured uncertainties. Except for ^226^Ra, the Victoreen 451P-DE-SI-RYR readings are always lower than those of the Thermo FH40G-L instrument. The differences in the exposure rate measurements from the calculations for ^60^Co are higher by approximately a factor of 5. Longer integration times for the Victoreen 451P-DE-SI-RYR are required to obtain more accurate measurements.

Source shielding adds an additional variable to the determination of the exposure rate values for the masking and target sources used for testing. Calculated values will depend on the choice of mass attenuation coefficients used to account for the source encapsulation. The calculated values for ^131^I and ^99m^Tc point sources surrounded by 7.64 cm of PMMA are shown in Table 5. The calculations assumed a PMMA density of 1.19 g/cm^3^ and the mass attenuation coefficients were extracted from [4]. The exposure rate constants calculated using MCNPX are approximately 2 times larger for ^131^I and 3.5 times larger for ^99m^Tc than the corresponding values obtained using the point source method. This is because the MCNPX method includes contributions from Compton scattered photons, while the point source method does not.

In order to obtain an estimate of the instrument behavior when measuring shielded sources, the ^133^Ba source from Table 2 and a ^57^Co source (Activity = 1.76 MBq (±10 %, k = 1), reference time = 9/1/12 12:00 PM EST) with the same type of encapsulation were shielded by placing them inside a PMMA container with a 9 cm wall thickness. The measurements and calculated exposure rate values are listed in Table 6. Measurements were performed at 0.2 m and 1 m. The ^57^Co and ^133^Ba sources were used instead of the ^99m^Tc and ^131^I because of their longer half-life and the similarity in their emitted gamma-ray energies.

For the ^57^Co source at 1 m from the detector, the readings were close to background levels, explaining the large variations observed in the measured values seen in Table 6. The measurements at 0.2 m agree within the measured uncertainties, given with a coverage factor of k = 2 (calculated as described for Table 4). The measured values at 0.2 m are approximately between 3 to 7 times larger than the calculated values using the point source method that does not account for Compton scattered photons. The data clearly demonstrate that the Compton scattered photons contribute significantly to the measured exposure rate or ambient dose equivalent rate in this source-detector geometry. Appropriate conversion factors should be applied to the measured ambient dose equivalent rates in order to compare them to calculated exposure rate values. ISO 4037-3 [11] provides a list of conversion factors for mono-energetic parallel photon beams that could be used to perform these calculations.

**Fig. 1:**
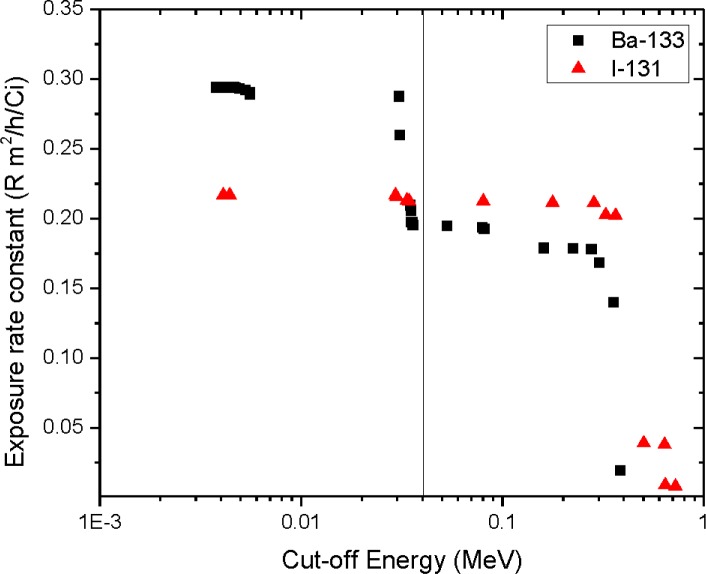
Calculated exposure rate constant as a function of cut-off energy for ^131^I and ^133^Ba.

Lastly, the exposure rate constants were calculated for each radionuclide as a function of the photon cut-off energy using the point source approximation. Figure 1 shows that calculated exposure rate constants for some radionuclides, such as ^131^I, are fairly insensitive to the choice of cut-off energy. However, the values for other radionuclides, such as ^133^Ba, show a much greater dependence. For instance, the exposure rate constant for ^133^Ba increases by more than 30 % when photons with energies less than 36 keV are included in the calculation. This result explains the variation in exposure rate measured by different detectors, the fundamental parameter being the thickness of the detector casing or sensitive window which determines the lowest photon energy that the instrument can measure.

## 5. Conclusions

The determination of the exposure rate values for testing radiation detection instruments against the ANSI/IEEE standards widely depends on the method used to determine the radiation field. As testing can occur in different laboratories, which may use different methods to determine the testing radiation field, the observed response of the instruments under test can be very different from laboratory to laboratory. These differences could cause an instrument to erroneously pass or fail an ANSI/IEEE test requirement depending on the laboratory that performs the testing. It is our recommendation that the ANSI/IEEE as well as the IEC standards define the test conditions using source activity or gamma-ray emission rate (for specific gamma-ray lines) instead of exposure rate or ambient dose equivalent rate values. This approach would help improve consistency in the fields used to evaluate radiation detectors across the various testing laboratories. Specifying the source activity or gamma-ray emission rates (for specific gamma-ray lines) with the associated allowed testing range (± 20 % from nominal activity value) has two key advantages: (1) the uncertainty in the measurement is known; and (2) the instrument manufacturers know exactly the lowest activity range for which their instrument needs to be designed to detect and/or identify a given isotope. In contrast, if the test fields are specified in the standards in terms of exposure and ambient dose equivalent rates, then the actual fields produced at the various testing laboratories might vary by orders of magnitude depending on the tools they use to determine them (measurements or calculations).

## Figures and Tables

**Table 1:** List of published ANSI/IEEE and IEC standards

ANSI/IEEE standards	IEC standards
ANSI/IEEE N42.32 Performance Criteria for Alarming Personal Radiation Detectors for Homeland Security	IEC 62401 Radiation protection instrumentation - Alarming personal radiation devices (PRD) for detection of illicit trafficking of radioactive material
ANSI/IEEE N42.33 Portable Radiation Detection Instrumentation for Homeland Security	IEC 62533 Radiation protection instrumentation - Highly sensitive handheld instruments for photon detection of radioactive material
ANSI/IEEE N42.34 Performance Criteria for Hand-held Instruments for the Detection and Identification for Radionuclides	IEC 62327 Radiation protection instrumentation - Hand-held instruments for the detection and identification of radionuclides and for the indication of ambient dose equivalent rate from photon radiation
ANSI/IEEE N42.35 Evaluation and Performance of Radiation Detection Portal Monitors	IEC 62244 Radiation protection instrumentation - Installed radiation monitors for the detection of radioactive and special nuclear materials at national borders
ANSI/IEEE N42.38 Performance Criteria for Spectroscopy-Based Portal Monitors Used for Homeland Security	IEC 62484 Radiation protection instrumentation - Spectroscopy-based portal monitors used for the detection and identification of illicit trafficking of radioactive material
ANSI/IEEE N42.43 Performance Criteria for Mobile and Transportable Radiation Monitors Used for Homeland Security	No IEC standard currently available for mobile and transportable systems
ANSI/IEEE N42.48 Performance Requirements for Spectroscopic Personal Radiation Detectors (SPRDs) for Homeland Security	IEC 62618 Radiation protection instrumentation - Spectroscopy-based alarming Personal Radiation Detectors (SPRD) for the detection of illicit trafficking of radioactive material
ANSI/IEEE N42.53 Performance Criteria for Backpack Based Radiation Detection Systems Used for Homeland Security	IEC 62694 Radiation protection instrumentation - Backpack-type radiation detector (BRD) for detection of illicit trafficking of radioactive material
No ANSI/IEEE standard currently available for highly sensitive neutron detectors	IEC 62534 Radiation protection instrumentation - Highly sensitive handheld instruments for neutron detection of radioactive material

**Table 2:** Activities for gamma-ray and neutron sources

Source	Activity (kBq)	Reference time
^241^Am	1910	6/15/2005 6:00 AM EST
^133^Ba	5480	6/15/2005 6:00 AM EST
^60^Co	1660	7/11/2011 12:00 PM EST
^137^Cs	3150	6/15/2001 6:00 AM EST
^226^Ra	295	3/7/2005 12:00 PM EST
^232^Th	550	3/1/2005 12:00 PM EST
^232^U	501	8/1/2006 12:00 PM EST

The uncertainty in the activities and neutron emission rate is 10 % (1 σ).

**Table 3:** Summary of calculated exposure rate constants for bare sources

Source	Exposure rate constant (R m^2^ h^−1^ Ci^−1^)					
	Point source method (1 keV)	Point source method (40 keV)	MCNPX method (LNHB)	MCNPX method (NBL)	Smith *et al.* [9]	Rad Pro Calculator [10]
^241^Am	0.0925	0.0155	0.1189	0.0970	0.0749	0.0166
^133^Ba	0.2941	0.1948	0.3045	0.3060	0.3041	0.1993
^60^Co	1.2942	1.2942	1.2956	1.2964	1.2907	1.2926
^137^Cs	0.3574	0.3523	0.3263	0.3269	0.3428	0.3211
^131^I	0.2169	0.2125	0.2210	0.2212	0.2199	0.2035
^40^K	0.0733	0.0733	0.0769	0.0802	0.0780	0.0779
^226^Ra	0.9415	0.7787	1.0410	0.8971	0.0039	0.7331
^99m^Tc	0.0672	0.0515	0.0777	0.0773	0.0795	0.0777
^232^Th	1.4386	1.1280	1.4811	1.1504	0.0144	0.0001
^232^U	0.6855	0.6855	-	0.7012	0.0234	0.0066

**Table 4:** Measured and calculated exposure rate values at 1 m for different encapsulated sources

Source	Measured values at 1 m						Calculated values at 1 m	
	Victoreen 451P-DE-SI-RYR (µSv/h)		Thermo FH40G-L (µR/h)		Ludlum 9DP (µR/h)		Point source method (40 keV) (µR/h)	MCNPX method (LNHB) (µR/h)
	Rate	Integration	Rate	Integration	Rate	Integration		
^241^Am	0.014 ± 0.027	0 ± 0.05	0.73 ± 0.09	1.15 ± 0.38	2.78 ± 0.36	1.26 ± 0.25	0.626	0.560
^133^Ba	0.261 ± 0.038	0.12 ± 0.05	31.7 ± 3.6	20.1 ± 2.4	20.9 ± 1.2	21.1 ± 0.8	13.6	20.1
^60^Co	0.506 ± 0.062	0.51 ± 0.06	65.3 ± 5.8	63.9 ± 5	48.1 ± 5.2	46.9 ± 1.8	47.6	47.5
^137^Cs	0.284 ± 0.050	0.24 ± 0.05	29.5 ± 2.4	27.4 ± 3.2	24.0 ± 2.9	24.2 ± 2.8	25.2	22.9
^226^Ra	0.089 ± 0.033	0.074 ± 0.037	5.53 ± 1.78	9.29 ± 1.89	6.37 ± 0.45	5.82 ± 0.73	6.10	7.01
^232^Th	0.131 ± 0.047	0.098 ± 0.051	25.7 ± 1.9	22.1 ± 1.8	11.3 ± 1.5	14.5 ± 1.5	16.9	17.2
^232^U	0.104 ± 0.030	0.12 ± 0.05	15.3 ± 1.6	14.3 ± 1.8	9.38 ± 1.82	7.98 ± 0.68	9.15	9.43

**Table 5:** Summary of calculated exposure rate constants for shielded sources

Source	Exposure rate constant (R m^2^ h^−1^ Ci^−1^)			
	Point source method (1 keV)	Point source method (40 keV)	MCNPX method (LNHB)	MCNPX method (NBL)
^131^I	0.0819	0.0817	0.1550	0.1554
^99m^Tc	0.0135	0.0134	0.0455	0.0457

**Table 6:** Measured exposure rate values for shielded sources

Source	Exposure rate and ambient dose equivalent rate values					
	Victoreen 451P-DE-SI-RYR (µSv/h)		Thermo FH40G-L (µR/h)		Point source method (40 keV) (µR/h)	
	at 0.2 m	at 1 m	at 0.2 m	at 1 m	at 0.2 m	at 1 m
^133^Ba	5.0 ± 0.6	0.144 ± 0.06	387 ± 98	8 ± 1	123	4.93
^57^Co	0.57 ± 0.10	0.002 ± 0.001	43 ± 6	0.55 ± 0.04	8.1	0.32

## Footnotes

1 NIST does not endorse the use of non-SI units. This paper uses non-SI units because it addresses the requirements listed in the ANSI/IEEE published standards.

2 Certain commercial equipment, instruments, or materials are identified in this paper to foster understanding. Such identification does not imply recommendation or endorsement by the National Institute of Standards and Technology, nor does it imply that the materials or equipment identified are necessarily the best available for the purpose.
